# Metagenome sequencing-based strain-level and functional characterization of supragingival microbiome associated with dental caries in children

**DOI:** 10.1080/20002297.2018.1557986

**Published:** 2018-12-28

**Authors:** Nezar Noor Al-Hebshi, Divyashri Baraniya, Tsute Chen, Jennifer Hill, Sumant Puri, Marisol Tellez, Nur A. Hasan, Rita R. Colwell, Amid Ismail

**Affiliations:** a Oral Microbiome Research Laboratory, Maurice H. Kornberg School of Dentistry, Temple University, Philadelphia, PA, USA; b Department of Microbiology, Forsyth Institute, Cambridge, MA, USA; c Department of Pediatric Dentistry and Community Oral Health Sciences, Maurice H. Kornberg School of Dentistry, Temple University, Philadelphia, PA, USA; d CosmosID Inc., Rockville, MD, USA; e University of Maryland Institute for Advanced Computer Studies, University of Maryland, College Park, MD, USA; f Maryland Pathogen Research Institute, University of Maryland, College Park, MD, USA

**Keywords:** Bacteria, dental caries, High-throughput nucleotide sequencing, microbiota, metagenome

## Abstract

Studies of the microbiome associated with dental caries have largely relied on 16S rRNA sequence analysis, which is associated with PCR biases, low taxonomic resolution, and inability to accurately study functions. Here, we employed whole metagenome shotgun sequencing, coupled with high-resolution analysis algorithm, to analyze supragingival microbiomes from 30 children with or without dental caries. A total of 726 bacterial strains belonging to 406 species, in addition to 34 bacteriophages were identified. A core bacteriome was identified at the species and strain levels. Species of *Prevotella, Veillonella*, as yet unnamed *Actinomyces*, and *Atopobium* showed strongest association with caries; *Streptococcus* sp. AS14 and *Leptotrichia* sp. Oral taxon 225, among others, were overabundant in caries-free. For several species, the association was strain-specific. Furthermore, for some species, e.g. *Streptococcus mitis* and *Streptococcus sanguinis*, sister strains showed differential associations. Noteworthy, associations were also identified for phages: *Streptococcus* phage M102 with caries and *Haemophilus* phage HP1 with caries-free. Functionally, potentially relevant features were identified including urate, vitamin K2, and polyamine biosynthesis in association with caries; and three deiminases and lactate dehydrogenase with health. The results demonstrate new associations between the microbiome and dental caries at the strain and functional levels that need further investigation.

## Introduction

Over the past 15 years, Sanger sequencing of 16S rRNA clones, frequently coupled with reverse-capture checkboard DNA-DNA hybridization, has been widely employed to study the microbial community associated with dental caries [1–10]. Results of these studies have revealed significant diversity, and many novel species/phylotypes have been identified. In addition to substantiating evidence for the role of mutans streptococci and lactobacilli, they quite consistently revealed an association between a number of microorganisms and dental caries, including *Propionibacterium* spp., *Bifidobacterium* spp., *Veillonella* spp., *Actinomyces* spp. and *Atopobium* spp., as well as acidogenic non-mutans streptococci, especially in those subjects for which *Streptococcus mutans* was not detectable. It has also been possible to identify candidate health-associated bacterial species, such as *Streptococcus mitis*. Notably, most of these studies focused on caries of primary teeth [2,4,5,7,9,10].

One drawback of Sanger sequencing of 16S rRNA clones, however, has been its high cost and the laborious laboratory work required, limiting the number of samples and clones that can be feasibly analyzed [11]. Fortunately, this limitation has been overcome by the advent of next generation sequencing (NGS). NGS allows analysis of microbial communities to unprecedented depth and breadth at relatively lower cost. Thus, it offers an invaluable tool for analysis of the oral microbiome in health and disease [11]. Typically, studies using NGS to characterize microbial communities target one or more regions of the16S rRNA gene, which due to their hypervariability serve as good markers of bacterial taxa in samples. This approach has recently been used in a series of studies to explore microbiomes of dental caries [12–15], providing better insight into the diversity of the microbial community associated with dental caries. And despite methodological differences among these studies in terms of sampling (saliva vs. supragingival plaque vs. carious dentine), hypervariable regions selected for sequencing and the bioinformatic analysis pipeline used, a number of taxa consistently showed association with dental caries, including, *S. mutans, Lactobacillus* spp., *Propionibacterium* spp., *Veillonella* spp., and *Atopobium* spp.

Targeted 16S rRNA gene sequencing, however, requires gene amplification by PCR, which is known to introduce errors such as nucleotide substitution, insertion and deletion as well as chimera formation, leading to detection of spurious species and inflating microbial diversity [16,17]. In addition, PCR is beset by a number of biases such as 1) limited primer coverage, which can result in failure to amplify some taxa, particularly novel ones [18], and 2) differential amplification of templates, which can alter the relative abundance of species and thus distort the original microbial community structure [19]. Shotgun whole metagenome sequencing (WMS) does not involve gene amplification by PCR and allows identification of microbial taxa comprising a community to a higher resolution than 16S rRNA-based sequencing; it also enables exploring microorganisms other than bacteria, e.g. viruses and fungi [20]. In addition, WMS data can be analyzed to characterize the functional potential of the microbial community (gene and pathway analysis).

In the pioneer study by Belda-Ferre et al. [21], WMS was employed to characterize the functional potential of the supragingival microbiome; in addition, 16S rRNA sequences extracted from the data were used to obtained taxonomic profiles. However, no differential abundance analysis was performed, probably because of the small sample size (four subjects with caries and two without). In a larger-scale study, Belstrøm et al. combined metagenomics and metatranscriptomics to study the oral microbiome of 10 subjects with healthy mouth, 10 with dental caries and 10 with periodontitis [22]. Consistent with the literature, the study showed mutans streptococci, *Lactobacillus* spp. and *Veillonella* spp., and their transcripts, to be associated with dental caries. It is unfortunate, however, that saliva rather than supragingival plaque was used for the analysis, which may explain why functional analysis did not return relevant results.

So far, there has been no attempt to exploit WMS data to obtain strain-level taxonomic assignments, explore association with microorganisms other than bacteria or perform in depth functional analyses. The aim of this study, therefore, was to employ WMS to perform strain-level, multi-kingdom profiling as well as comprehensive functional characterization of the supragingival microbiome associated with dental caries in children.

## Materials and methods

### Study subject recruitment

The study was conducted in compliance with the Helsinki Declaration on medical research involving human subjects and was approved by the Temple University’s Institutional Review Board (protocol # 24355). An assent was obtained from each participating child and an informed written consent was obtained from his/her parent or guardian.

Study children were recruited from the Pediatric Dentistry Clinic at the Temple University Kornberg School of Dentistry. Each child had to fulfill the following criteria: 6–10 years old with all first permanent molars erupted (mixed dentition); no history of antibiotic, antifungal, or steroid intake or use of mouthwashes in the three months prior to sampling; no evidence of oral abscess or candidiasis; no history of diabetes, immunodeficiency, or dental prophylaxis in the previous 30 days. Supragingival plaque samples were obtained from eligible children, as described below, before full mouth prophylaxis, and clinical examination were performed. Caries status was assessed with clinical visual examination following the International Caries Classification and Management System (ICCMS) [23] as well as radiographic examination. Eventually, 10 caries-free children (defined as having no carious lesions, including white spots, and no previous fillings), 10 with early caries (defined as having at least one tooth with early, non-cavitated carious lesion), and 10 with advanced caries (defined as having at least one tooth with cavitated carious lesion) were recruited. The characteristics of the study groups are presented in Supplementary Table 1.

### Microbial sampling and DNA extraction

A whole-mouth, supragingival plaque sample was obtained from each study subject as follows: First, the sampling sites were isolated with cotton rolls and air-sprayed to minimize contamination with saliva. Then, a sterile curette was used to scrape dental plaque from the buccal surface of all teeth present. In subjects with active caries, cavities were avoided. As the sample was collected, the plaque was pooled by wiping it onto a single sterile gutta–percha point. Finally, the point with supragingival plaque sample was placed it into a tube containing sterile TE buffer and stored at −80°C.

At the time of DNA extraction, the samples were thawed and vortexed vigorously to dislodge plaque from the gutta–percha points into the buffer. Sterile forceps (a new pair for every sample) were used to remove the points from the tubes. Each plaque sample was pelleted by centrifugation at maximum speed, washed twice in 1 ml PBS, and digested by re-suspending in 250 μl PBS containing 25 μl Metapolyzme (Sigma, USA), and incubating at 37° for 3 h. The lysate was used for DNA extraction employing the ZymoBiomics miniprep kit (Zymo Research, Germany) according to the manufacturer’s instructions. Quantity and quality of the extracts were assessed using a Qubit 3.0 (ThermoFisher Scientific, USA).

### DNA sequencing and compositional analysis

Fragment libraries were prepared from 200 ng DNA, using the IonXpress Plus Fragment Library kit (ThermoFisher Scientific, USA), according to the manufacturer’s instructions. The libraries were sequenced on an Ion S5XL sequencer (ThermoFisher Scientific) to generate 200 bp single-end sequence reads. Each sample was sequenced, with an average of 21 M sequence read depth. Unassembled sequencing reads were directly analyzed with the CosmosID (originally called GENIUS) metagenomic software (CosmosID Inc., Rockville, MD), as described elsewhere [24,25] for multi-kingdom microbiome composition analysis and quantification of relative abundances at all taxonomic levels. Briefly, the system utilizes a high performance data-mining k-mer algorithm and highly curated dynamic comparator databases (GenBook®) comprising over 150,000 microbial genomes and gene sequences representing over 10,000 bacterial, 5,000 viral, 250 protists and 1,500 fungal species. It constitutes both publicly available genomes or gene sequences through NCBI- RefSeq/WGS/SRA/nr, PATRIC, M5NR, IMG, ENA, DDBJ, CARD, ResFinder, ARDB, ARG-ANNOT, mvirdb, VFDB etc., in addition to a subset of genomes sequenced by CosmosID and its collaborators.

The pipeline has two separable comparators. The first consists of a pre-computation phase and a per-sample computation. The input to the pre-computation phase is a reference microbial database, and its output is a whole genome phylogenetic tree, together with sets of variable-length k-mer fingerprints (biomarkers) that are uniquely identified with distinct branches, nodes and leaves of the tree. The second per-sample, computational phase searches the hundreds of millions of sequence reads against the fingerprint sets in minutes. The resulting statistics are analyzed to give fine-grain composition and relative abundance estimates at all branches, nodes and leaves of the tree. The second comparator uses edit distance-scoring techniques to compare a target sample with a reference set. Overall classification precision is maintained through aggregation statistics. The first comparator finds reads for which there is an exact match with a k-mer uniquely identified in one or a set of reference strains; the second comparator then statistically scores the entire read against the reference to verify that the read is indeed uniquely identified with that set. For each sample the reads from a species are assigned to the strain with the highest aggregation statistics.

The resultant taxa abundance tables were used to calculate observed and expected species richness, alpha diversity indices, and beta diversity distance matrices. At each taxonomic level, microbial taxa which were present in all the subjects, irrespective of their relative abundances, were considered as core microbiome. Principle Coordinate Analysis (PCoA) was performed to cluster samples based on abundance Jaccard distance matrix (community structure). Differentially abundant taxa at the species and strain levels were identified using Linear discriminant analysis Effect Size (LEfSe) [26], with ‘caries’ and ‘caries-free’ as classes and ‘early-caries’ and ‘advanced caries’ as subclasses.

### Functional analysis

Trimmomatic, v 0.36 [27] was used to quality-filter the raw reads: trimming with a quality sliding window of 4:8; cropping sequences to remove 20 bases from the start and bases beyond 220 from the end (based on results of analysis by FASTQC); and filtering out sequences less than 150 bp long. Reads were processed using Kneaddata (https://bitbucket.org/biobakery/kneaddata/wiki/Home) to remove human DNA sequences. Quality trimmed reads were processed through HUMAnN2 (http://huttenhower.sph.harvard.edu/humann2) with default settings, which includes 1) screening with MetaPhlAn2 for taxonomic identification using clade-specific markers [26]; 2) mapping reads to annotated pangenomes of the identified species in the ChocoPhlAn database using Bowtie2 [28] to obtain species-specific gene lists (nucleotide search); 3) translated search of unmapped reads against UniRef90 protein reference database [29] using DIAMOND [30]. The generated gene lists are then collapsed into protein families and enzyme classes/pathways using PFam [31] and MetCyc [32] databases, respectively. Species contribution is based on results obtained in step 2 above. The resulting gene families and pathway abundance files from all samples were joined and normalized to relative abundance. Merged data were unstratified and LEfSe was employed to identify differentially abundant features, as described above.

## Results

### Sequencing and data processing statistics

Ion torrent sequencing yielded an average of 21.1 million reads per sample (range 10–36 million reads). Raw reads (publically available at ftp://www.homd.org/publication_data/20180420/fastq/) were directly used for compositional analysis, but were quality-filtered for functional analysis. Use of Trimmomatic resulted in dropping an average of 31.41% of the reads from each sample. Kneaddata removed human DNA sequences, which accounted for 0.03−10.35% of the reads (average of 2.26%). Details of read counts for each sample, before and after each quality control step, are provided in Supplementary file 1. An average of 13.8 million reads per sample (median 12.9 million; range 7.7–27.7 million reads) was obtained for downstream analysis. In compositional analysis, 51.26 % of the reads per sample (on average) hit the k-mer markers in the database, while in functional analysis (HUMAnN2) an average 44.93% could be assigned a function (the translated search stage in HUMAnN2 only reports proteins with >50% coverage by default).

### Overall microbial profile

Bacterial sequences accounted for 99.6% of all reads that hit k-mer markers. The analysis pipeline resulted in identification of 726 bacterial strains belonging to 406 species, 94 genera, and 12 phyla, in addition to 34 bacteriophages, two protists and two fungi. The latter were identified in single samples, while further analysis of the protist sequences indicated potential false positives, so both fungi and protists are not discussed in additional detail.

**Figure 1.**
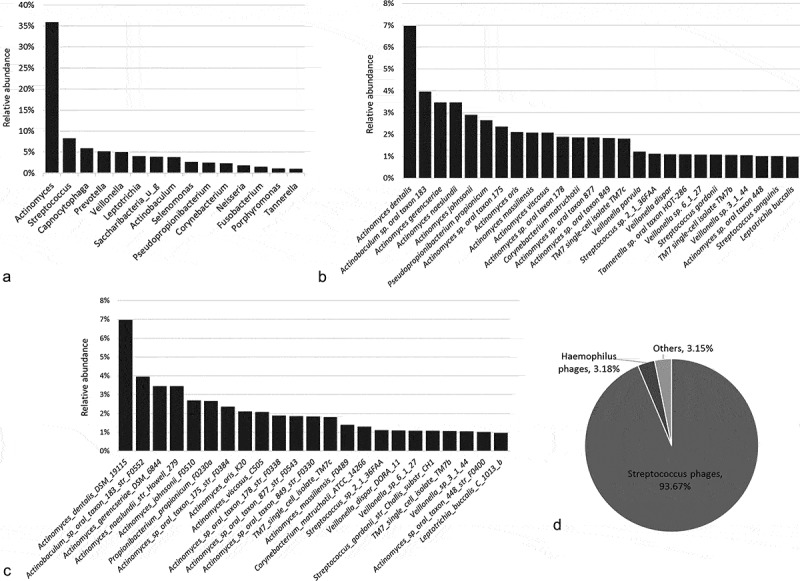
The microbiome profile of supragingival plaque. DNA extracted from supragingival samples was shotgun-sequenced on an Ion S5XL sequencer. The generated reads (200 bp) where classified to the strain level using the CosmosID metagenomic pipeline that employs clade-specific k-mers derived from a comprehensive reference genome database (see text for details). (a) bacterial phyla, (b) bacterial species and (c) bacterial strains identified at average relative abundance ≥ 1%. (d) Relative abundance of major groups of bacterial phages detected.

Sample relative abundances and detection frequencies for all bacterial phyla, genera, species, and strains detected in this study are presented in Supplementary files 2–5. At the phylum level, the core bacteriome comprised Actinobacteria (46.7%), Firmicutes (22.5%), Bacteriodetes (14.5%), Proteobacteria (5.8%), Fusobacteria (5.8%), Saccharibacteria (4%), and Spirochetes (0.54%). At the genus level, 42–61 genera were detected per subject. Figure 1(a) illustrates relative abundances of the major 15 genera identified (those present at ≥ 1% relative abundance). Together, they comprised 87% of the average bacteriome. *Actinomyces* alone accounted for 36.05%, followed by *Streptococcus* (8.4%) and *Capnocytophaga* (6.1%). These 15 genera were identified in all samples and, together with an additional 19 genera, comprised the core bacteriome (Supplementary file 6).

The number of bacterial species/strains per sample ranged from 217 to 301. The 28 species and 23 strains with average relative abundance ≥1% are shown in Figure 1(b, c). In total, they comprised 53% and 48% of the average bacteriome at the species and strain levels, respectively. The majority of them were a part of the core bacteriome, comprising 133 species and 96 strains (Supplementary files 7 and 8). Named *Actinomyces* spp. and strains were the most prominent of the core taxa, in addition to *Actinobaculum* sp. oral taxon 183 str F0552, *Pseudopropionibacterium propionicum* str F0230a, *Corynebacterium matruchotii* str ATCC 14266, *Veillonella parvula* str ACS 068 V Sch12, *Veillonella dispar* str DORA 11, *Leptotrichia buccalis* str *C1013b*, TM7 single isolates, TM7a and TM7b, and corresponding species. *Streptococcus gordoni* and *Streptococcus sanguinis* were among the top core species, but no single strain of either species was detected in all samples.

In addition to bacteria, 34 strains of bacteriophages were also identified (Supplementary file 9). *Streptococcus* phages accounted for 93.6 % of all phage sequences, while *Haemophilus* phages accounted for 3.18% (Figure 1(d)).

### Species richness and diversity

**Figure 2.**
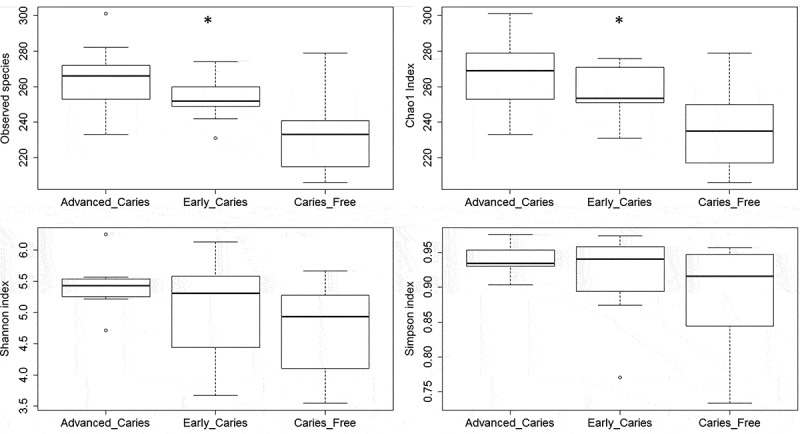
Species richness and alpha diversity. The taxonomic profiles obtained with compositional analysis of the sequences were used to calculate alpha diversity indices employing standard QIIME scripts. Significance of differences between groups were then sought with the Kruskal-Wallis test. The figure shows a comparison of observed species richness, Chao1 (expected richness), Shannon and Simpson indices between the study groups. * P ≤ 0.05.

**Figure 3.**
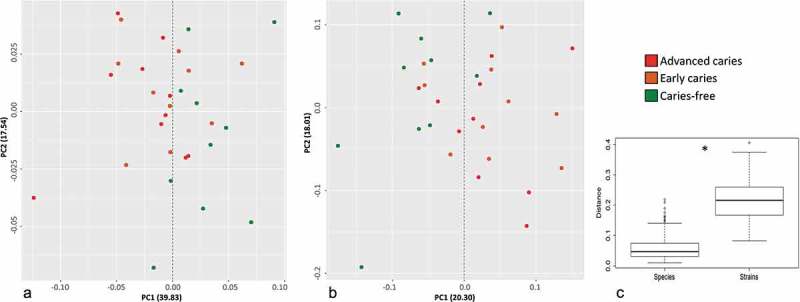
β-Diversity analysis. Inter-sample distances were calculated based on abundance Jaccard index employing standard QIIME scripts. The samples were then clustered using Principle Coordinate Analysis (PCoA) to visualize distances at the species (a) and strain (b) levels. (c) Quantitative representation with boxplots of distances between the samples based on abundance Jaccard index at the species and strain levels. * P ≤ 0.001, Wilcoxon rank sum test.

Results of comparison of species richness and alpha diversity between groups are shown in Figure 2. The caries groups had significantly higher observed species richness and Chao1 index (expected species richness); differences in Shannon and Simpson indices were not significant. In PCoA, caries and caries-free subjects formed separate clusters at both species and strain levels, but clustering by caries subclass, i.e. early and advanced caries, was not observed (Figure 3(a,b)). Inter-sample distances, however, were significantly higher at the strain level (Figure 3(c)).

### Differentially abundant species and strains

**Figure 4.**
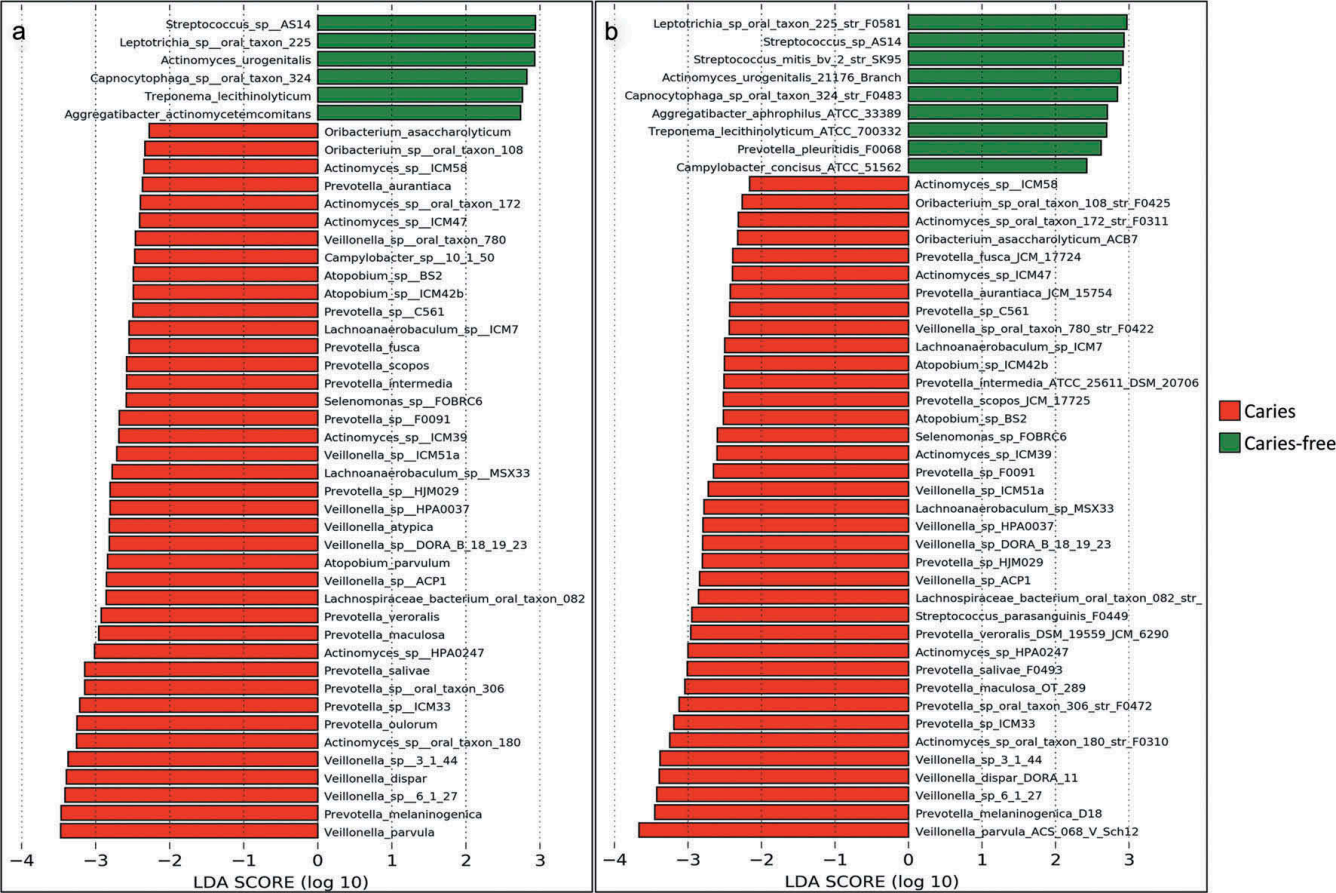
Differentially abundant taxa. (a) species and (b) strains that showed significant differences in relative abundance between the caries and caries-free groups, as identified by linear discriminant analysis (LDA) effect size analysis (LEfSe), with ‘caries’ and ‘caries-free’ as classes and ‘early-caries’ and ‘advanced caries’ as subclasses. Note: many unnamed species, e.g. *Streptococcus* sp_AS14, are represented in the database by only one strain, and in this case the species and strain names are identical.

Differentially abundant species and strains observed for the caries and caries-free groups are shown in Figure 4. Fourteen *Prevotella* spp., prominently *Prevotella melaninogenica*, 10 *Veillonella* spp., primarily *Veillonella parvula*, six unnamed *Actinomyces* spp., three *Atopobium*, and two *Oribacterium* spp. were found to be associated with dental caries, while only six species, including *Streptococcus* sp. AS14 and *Leptotrichia* sp. Oral taxon 225, were more abundant in caries-free subjects. Detailed plots showing selected differentially abundant features are presented in Supplementary Figure 1, demonstrating an association (or inverse association) with disease severity, i.e. lowest average abundance in caries-free and highest in advanced caries (or vice versa). Although *S. mutans* was not detected by LEfSe analysis, a separate analysis with Kruskal–Wallis test followed by multiple Wilcoxon test for pairwise comparisons (the basic statistics of LEfSe), revealed a significant difference only between the advanced caries and no caries groups (Supplementary Figure 2).

**Figure 5.**
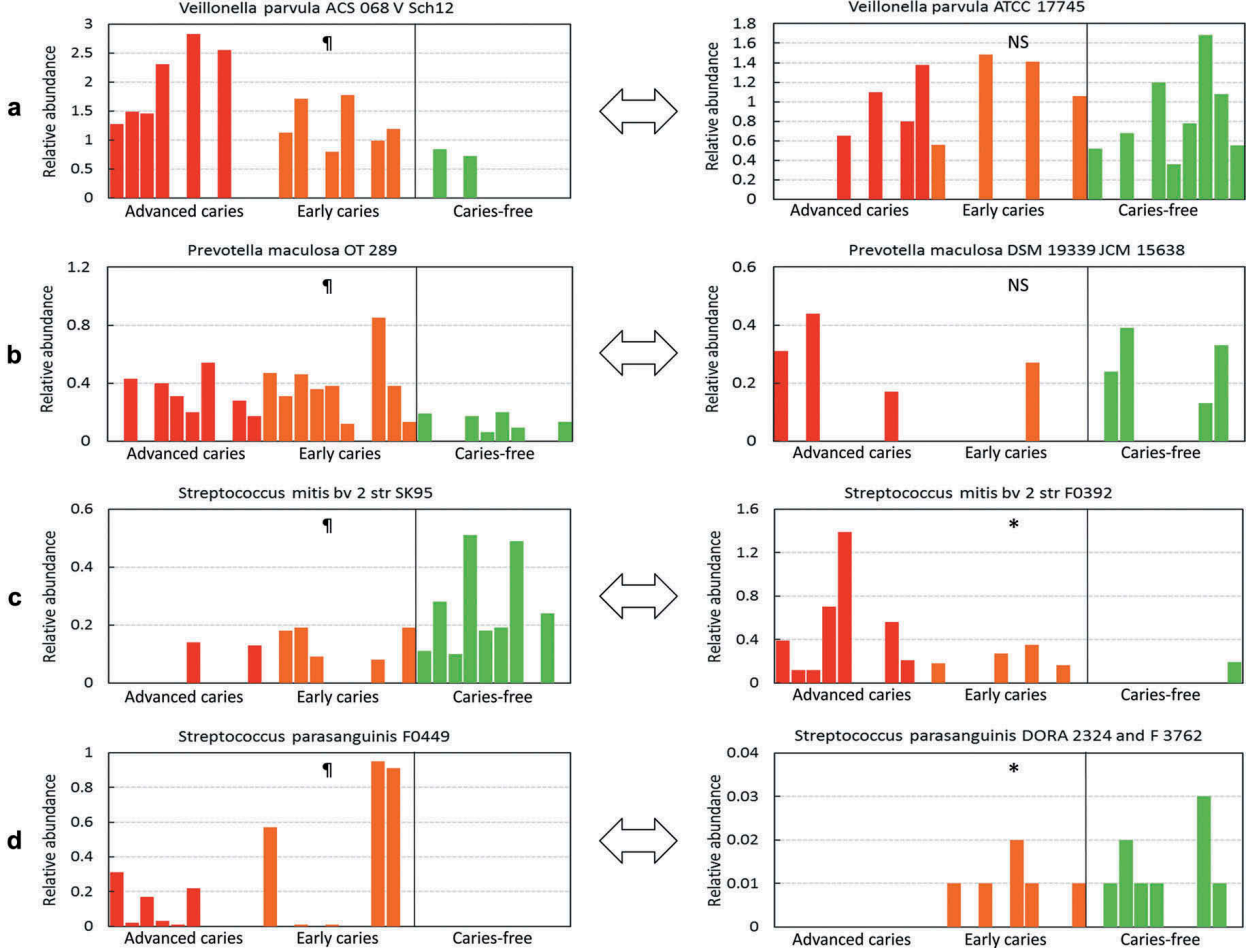
Differential association with health and disease at the strain level. Examples of strains belonging to same species but showing different patterns of association with the study groups. ¶Identified as differentially abundant by LEfSe (see Figure 4). For the right panel, significance of differences was sought with the Kruskal-Wallis test. NS: no significant difference; * P ≤ 0.05.

At the strain level, four different scenarios were identified. In one scenario, only one strain of a species was detected across all samples and it, therefore, showed similar association to that of the corresponding species. In another scenario, a species was represented by more than one strain, but no particular strain accounted for the species association, such as in the case of *Atopobium parvulum* and *Veillonella atypica*; i.e. there were no significant differences between groups at the strain level, despite association at the species level. In the third scenario, a species was represented by more than one strain, but only a specific strain showed significant association, e.g. strains ASC 068 V Sch12 and OT 298 of *V. parvula* and *Prevotella maculosa*, respectively (Figure 5(a,b)). In the fourth and most interesting scenario, strains belonging to the same species showed a differential association with health and disease. For example, *S. mitis* bv 2 str SK95 and *Streptococcus parasanguinis* str FO449 were found to be associated with caries and caries-free groups, respectively, but sister strains showed opposite associations, as shown in Figure 5(c,d), with no association at the species level.

No significant differences in bacteriophage relative abundance was observed between groups. However, Chi-square analysis showed *Streptococcus* phage M102 to be significantly more prevalent – actually exclusively so – in the caries groups, while *Haemophilus* phage HP1 was detected at significantly higher rates in the caries-free group (Supplementary Figure 3).

### Differentially abundant functional attributes

**Figure 6.**
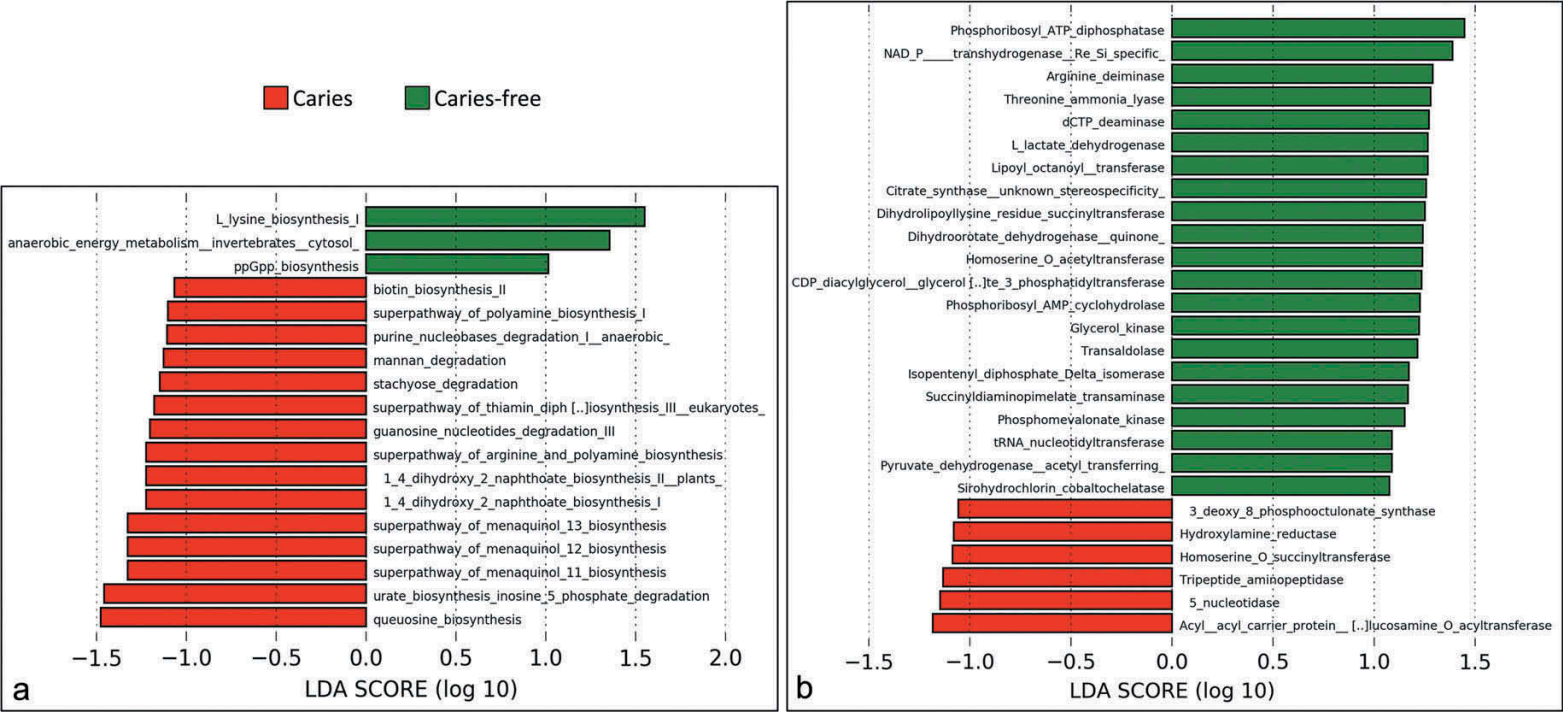
Differentially enriched microbial features. Quality-filtered sequences were functionally analyzed with HUMAnN2, which involves nucleotide search by mapping to pangenomes in ChocoPhlAn and translated search against Uniref90. Generated gene lists are collapsed into protein families, class families and pathways using Pfam and Metacyc databases. Linear discriminant analysis (LDA) effect size analysis (LEfSe) was then used to identify pathways (a) and enzyme classes (b) with significant differences in relative abundance between the caries and caries-free groups.

**Figure 7.**
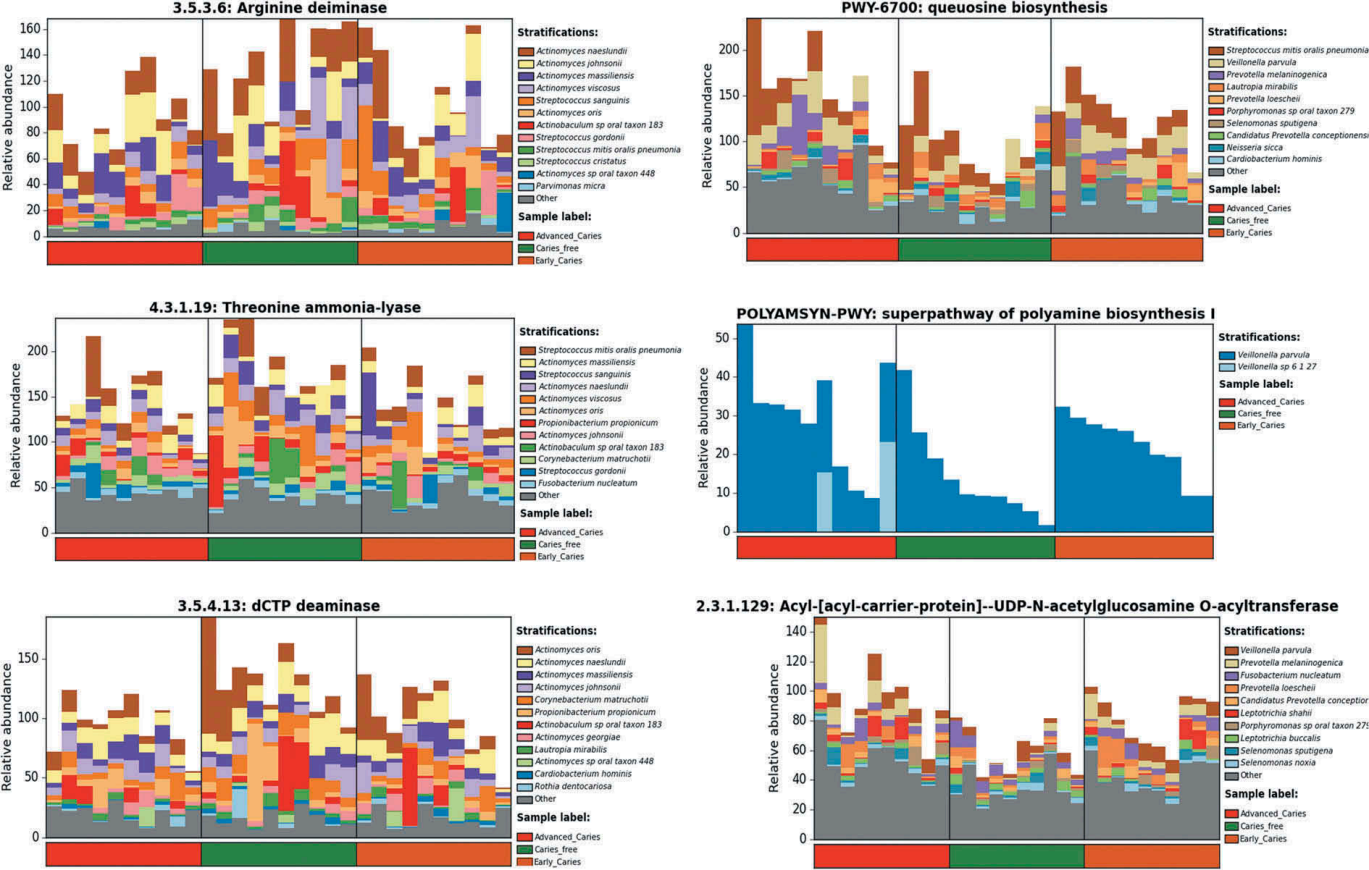
Species contributions to key functions. For each identified enzyme class or pathway, HUMAnN2 calculates species contribution based on results from nucleotide search in which reads are mapped to annotated pangenomes of known species. In the figure are species contributions to six features of potential relevance to dental caries.

Biosynthesis of queuosine, urate, 1,4-dihydroxy-2-naphthoate, menaquinols, and polyamine comprised the most common pathways associated with caries, while L- lysine biosynthesis was strongly associated with caries-free (Figure 6(a)). Potentially relevant enzyme classes differentially abundant between groups included arginine, threonine, and dCTP deiminases, as well as lactate dehydrogenase in association with health and acyl-acyl carrier protein and 5ʹ-nucleotidase with caries (Figure 6(b)). Sulfatase was the only protein family overrepresented in the caries groups (Supplementary Figure 4), while proteins involved in signal transduction, transcriptional regulation and membrane transport were enriched in the caries-free group. Figure **7** shows those species linked with selected differentially abundant functional attributes. The *Actinomyces* spp. not associated with dental caries, including *A. naeslundii, Actinomyces massiliensis, Actinomyces johnsonii, Actinomyces viscosus*, and *Actinomyces oris*, in addition to *S. mitis, S. sanguinis* and *C. matruchotii*, were major contributors to the three deiminases. *V. parvula* and *Veillonella* sp. 6127 were the sole contributors to polyamine synthesis and, along with *S. mitis* and *P. melaninogenica*, also contributed to queuosine biosynthesis.

## Discussion

To the best of our knowledge, this is the first study to characterize the supragingival microbiome associated with dental caries at the strain level using WMS. The CosmosID analysis pipeline employs clade-specific markers with strain-level resolution, generated from a large curated database (150,000+ genomes and gene sequences) [24,25]. In a recent study comparing 11 metagenomic analysis tools, BlastMegan was reported to provide the best overall performance [33]. Using the same training sets employed in that study, we found CosmosID to outperform BlastMegan, especially at the sub-species level (http://www.cosmosid.com/blog-cosmosid/benchmarking-genome-biology-2017). However, regardless of what analysis tool is used, false positives cannot be completely eliminated. In addition, the analysis assigns sequences of a species in a given sample to the most significant strain, resulting in identification of a single strain per species, and in turn, obscuring strain diversity within a sample, a limitation that is being addressed in the coming version of the analysis pipeline. Nevertheless, the identified strains are probably the predominant ones. In fact, a recent study of the gut microbiome showed that, in most cases, each species was either exclusively represented or dominated by a single strain [34]. Consistently, previous studies of dental caries have found that while children usually carry more than one genotype of *S. mutans*, a single genotype is generally predominant [35,36].

In the caries groups, we sampled the supragingival microbiome from surfaces unaffected by caries, with the assumption there would be a minimal effect of the disease process and that any alterations identified would represent early driver events that can serve as targets for prevention. In contrast, microbial changes within the dental caries lesions are late events and more likely a result rather than cause of disease. However, since sampling was performed prior to clinical examination, and despite extreme care, the possibility that plaque from surfaces with early carious lesions was accidentally collected cannot be totally excluded and is one limitation of this study. Nevertheless, since plaque was pooled from mostly intact surfaces, the effect of any contamination would have been diluted.

In contradiction with several previous reports [5–7,14], species richness and diversity, as observed in this study, were higher in the caries groups compared to the caries-free group. This is counterintuitive since the cariogenic process probably selects for acidogenic and aciduric species, leading to reduced diversity. However, our results may be explained by the fact that we did not sample the carious lesion itself, the site that particularly shows statistically significant lower diversity in previous studies [5,6]. Indeed, Belstrøm et al. analyzing saliva samples with WMS, found no significant differences in richness and diversity between health subjects and those with dental caries [22].

The current study, however, substantiates existing evidence for the association between several species other than *S. mutans* and dental caries, including, *Veillonella* spp., *Atopobium* spp., and *Actinomyces* spp. For the latter, as yet unnamed species were found to be associated with caries, while recognized species such as *A. naeslundii* tended to be associated with health, consistent with previous studies [1,4]. We found that *Prevotella* spp. also showed significant association with caries, which substantiates results from a previous longitudinal study in which *Prevotella*, but not *S. mutans* was found to be the main predictor of early childhood caries [37]. In fact, *S. mutans* in the current study was identified in very low abundance and showed significant association with advanced caries only. Consistently, Simǒn-Soro et al. found that despite a significant increase in the proportion of *S. mutans* with progression of caries, non-mutans streptococci were far more abundant in the carious lesions [38]. These findings evoke the current debate that questions its primary role in dental caries [39,40]. There is increasing evidence to support that dental caries results from a dysbiosis involving different oral microbial taxa instead of the activity of a single taxon [41].

*Streptococcus* sp. AS14 and *Leptotrichia* sp. Oral taxon 225 showed the strongest association with health. *Leptotrichia* spp. were found to be overrepresented in heath in a previous study [1], although the association appears to vary from one species to another [9,10]. *Streptococcus* sp. AS14 is a human isolate, described here for the first time to occur in the oral cavity. Its potential role in dental caries merits further investigation. The most intriguing results involved *S. mitis* and *S. parasanguinis*, for which sister strains showed differential associations, which likely explains the controversy concerning the role of these species in the literature, with results of some studies showing association with caries and others reporting association with health [4,5,7,9]. In this study, *Streptococcus* phage M102 and *Haemophilus* phage HP1 correlated with caries and health, respectively, another novel finding. Interestingly, *Streptococcus* phage M102 is specific for *S. mutans* serotype C [42], the most common serotype of *S. mutans*. Given that the phage and its host were co-detected, the former may have occurred as prophage. Thus, *Streptococcus* phage M102 may act as a marker for *S. mutans* serotype C and could then be targeted to induce lysis of *S. mutans*, a potential prevention strategy of dental caries that is worth exploring.

The arginine, threonine and dCTP deiminases genes were found to be overrepresented in the caries-free group. Deiminase activity results in release of ammonia, which plays an important role in prevention against caries by neutralizing acids [43]. In line with this, a recent proteomic study found arginine deiminase to be enriched in dental plaque from caries-free individuals [44]. In this study, members of *Actinomyces* spp. especially *A. naeslundii, A. johnsonii* and *A. massiliensis*, in addition to *S. mitis* and *S. sanguinis*, were found to be major contributors to the three ammonia generating enzymes (Figure 7). Arginolytic activity of some of these species has previously been demonstrated [43] . However, threonine and dCTP deiminases have not been previously implicated in dental caries. The lactate dehydrogenase (LDH) gene was also enriched in caries-free samples, which again concurs with results from the same proteomic study referred to above. Interestingly, a historical study found LDH to delay onset and reduce severity of caries in rats [45]. L- lysine biosynthesis was also over-abundant in caries-free subjects. Interestingly, this amino acid was reported in a much earlier study to inhibit biofilm formation by *S. mutans* in vitro [46].

The pathways, enzymes, and protein families that were found to be overrepresented in the caries group are potentially relevant to the cariogenic process. Polyamines, for example, are known to play an important role in biofilm formation by many bacteria [47] and may thus have a similar role in formation of dental plaque. 1,4-dihydroxy-2-naphthoate and menaquinols are precursors of vitamin K2 (menaquinone) which, in a recent study, has been shown to enhance biofilm formation by *Staphylococcus aureus* [48]. Similarly, there is evidence to suggest uric acid boosts biofilm formation by *Enterococcus faecalis* [49]. 5ʹ-nucleotidase is also potentially important as 5ʹ-nucleotidase inhibitors have been found to inhibit growth and glucan formation by *S. mutans* [50]. It would be interesting to explore the role of these compounds in biofilm formation by oral bacteria.

Queuosine-mediated modification of tRNA is involved in many cellular processes including signaling pathways and virulence of bacteria [51] and may contribute to carcinogenicity of dental plaque by downregulating arginine deiminase expression [52]. Sulfatases were also found to be enriched in the caries groups. Interestingly, according to an old theory, bacterial sulfatases hydrolyze sulfates of enamel and dentin, which in turn results in sulfuric acid production [53]. Although, this theory is not accepted today, it is possible that sulphatases are involved in a different way. Some oral streptococci, for example, have been reported to possess mucin-sulfatase activity, which may result in abating the protective action of salivary mucin [54]. It should be emphasized, however, that functional results obtained by analysis of WMS data are predictive and should thus be interpreted cautiously.

Two studies have previously employed WMS to assess the microbiome associated with dental caries [21,22]. In the study by Belstrøm et al. [22], saliva rather than supragingival plaque samples were analyzed so a direct comparison would not be meaningful. The study, in any case, substantiated evidence for the classical cariogens, i.e. mutans streptococci and lactobacilli. The study by Belda-Ferre et al. [21] performed compositional analysis based on analysis of 16S rRNA reads extracted from the WMS data. Without adjustment for 16S rRNA copy numbers (e.g. three for *Actinomyces* and five on average for streptococci) a reliable comparison of compositional results cannot be made.

Two general microbiological findings, irrespective of dental health status, are worth elaboration. One is that *Actinomyces* spp. and strains, hence phylum Actinobacteria, were the most abundant taxa in the children’s supragingival plaque samples. This finding is inconsistent with results from previous studies that used universal 16S rRNA amplification (sequencing or reverse-capture DNA-DNA hybridization), and in which *Streptococcus* and *Veillonella* spp. and, subsequently phylum Firmicutes, were found to dominate [2,5,6,9,55]. On the other hand, studies based on PCR-independent technologies, namely checkboard DNA-DNA hybridization, reported relative abundance of *Actinomyces* to be as high as 63% [10,56]. This is also consistent with early culture studies showing a high abundance of *Actinomyces*, especially in mature plaque [57]. In fact, direct comparison of culture and clonal analysis of 16S rRNA showed Actinobacteria to be underrepresented when the latter method was employed [8]. This observation strongly indicates that estimates of relative abundances using WMS are more reliable than those obtained using 16S rRNA sequencing. Choice of method for DNA extraction also must be considered in accounting for differences between studies, as it has been shown to significantly influence microbial profiling [58] . In the current study, an enzymatic mixture (metapolyzyme) and bead beating were used to achieve lysis for maximum recovery of DNA from the different bacterial species in the samples; the average DNA yield was 113 ng/μl.

Another noteworthy observation is that the viral sequences (predominantly phages) were the only non-bacterial microbial sequences consistently detected in all samples, a finding which is in agreement with previous reports that identified bacteriophages as a resident population of the oral cavity. However, their role remains poorly understood [59]. Although, a complex fungal community has been described in the oral cavity [60], fungal sequences were identified in only 2 of the 30 samples analyzed. This may be explained by two possible reasons. The first is that fungi have no affinity for supragingival plaque. The second is that they were present at such a low abundance that they were not detected at the sequencing depth used, another potential limitation of the current study.

In conclusion, this study demonstrates the potential of WMS, coupled with robust analysis tools, to characterize the oral microbiome to high taxonomic resolution and obtain reliable estimates of relative abundance of taxa with accurate prediction of microbial community function. It also highlights the importance of assessing the relationship of the microbiome with oral diseases to the level of the strain, by showing how different strains within the same species may differ in their association with dental caries. These inter-strain differences can be exploited for preventive strategies, such as replacement therapy. Similarly, functional analysis identified several microbial attributes with relevance to the cariogenic process and these represent potential targets for intervention, for example by boosting health-associated microbial activities and/or interfering with disease-associated activities. The potential role of phages represents an additional avenue for caries prevention research. It remains, however, important to confirm and validate results from this study in a larger-scale study using a real functional approach such as metatranscriptomics.
