# Supplementary material for: Metagenome sequencing-based strain-level and functional characterization of supragingival microbiome associated with dental caries in children
Source: J Oral Microbiol. 2018 Dec 28;11(1):1557986. doi: 10.1080/20002297.2018.1557986 (PMC6327923; doi:10.1080/20002297.2018.1557986)
Supplement: Supplemental Material [file ZJOM_A_1557986_SM3789.zip › Supplemental data/Suppl. figures and tables - R1.docx]

Metagenome sequencing-based strain-level and functional characterization of supragingival microbiome associated with dental caries in children

Nezar Noor Al-hebshi, Divyashri Baraniya, Tsute Chen, Jennifer Hill, Sumant Puri, Marisol Tellez, Nur Hassan, Rita R. Colwell and Amid Ismail

**Supplementary materials**

**Supplementary Table 1. Characteristics of the study subjects**

| **Variable** | **Caries free (n=10)** | **Early caries**  **( n=10)** | **Advanced caries**  **( n=10)** |
| --- | --- | --- | --- |
| **Age** (mean±SD) ^*^ | 8.6±1.07 | 8 ±13.1.63 | 8.2 ±1.4 |
| **Gender:**  Male  Female | 50%  50% | 30%  70% | 40%  60% |
| **Race:**  African American  Caucasian | 80%  20% | 100%  0% | 80%  20% |
| **# Carious lesions** (mean±SD) | 0 | 1.70±2.11 | 2.7±1.05 |
| **DNA yield** (mean±SD) ^*^ | 83.7±33.57 | 144.5±85.1 | 112.4±54.8 |

* Differences insignificant by Kruskal-Wallis test.


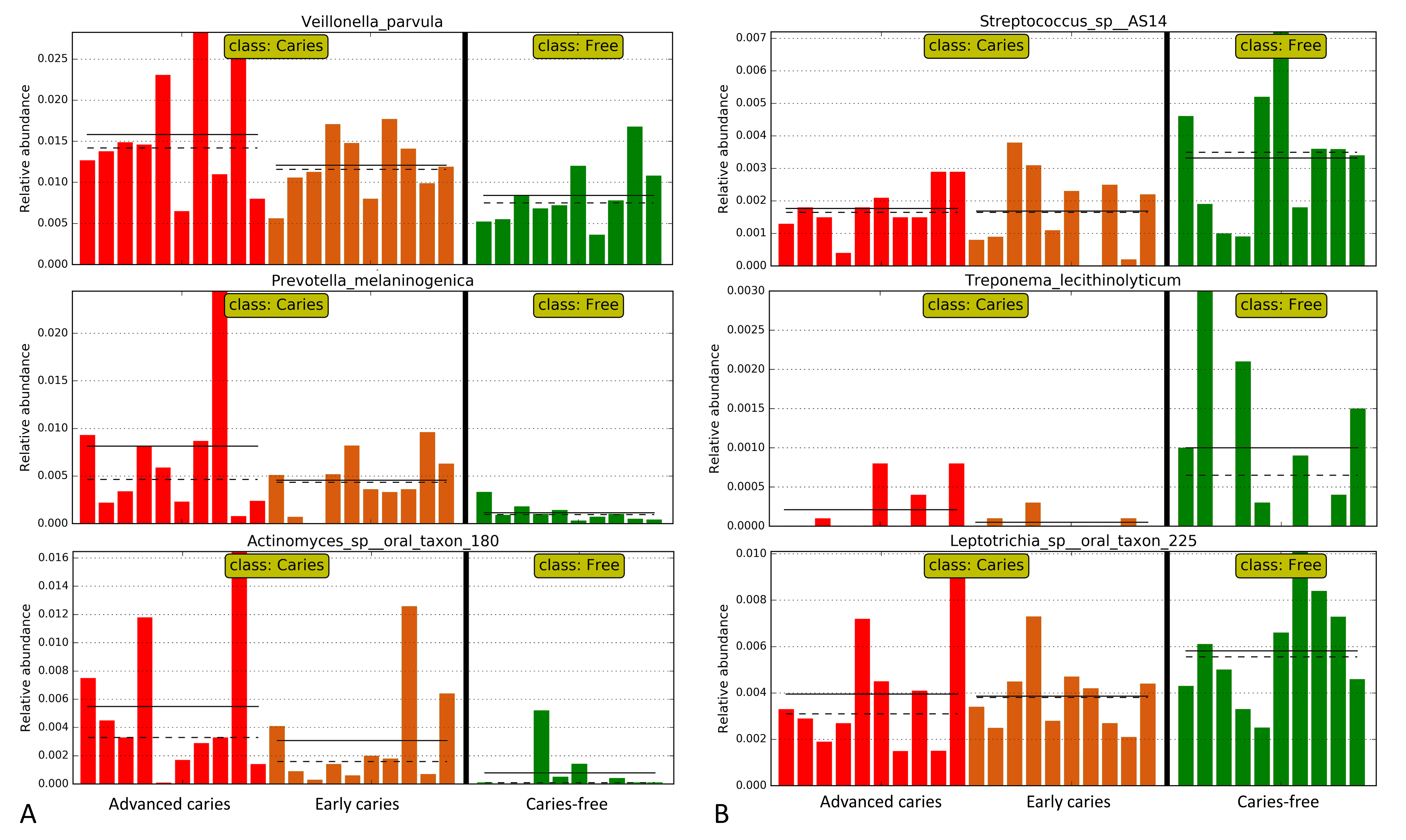


**Supplementary Figure 1.** Detailed plots with per sample, subclass and class data for six selected bacterial species that have been identified as differentially enriched by LEfSe (See **Figure 4** in the main article). Straight and dotted lines correspond to the mean and median of the subclasses, respectively


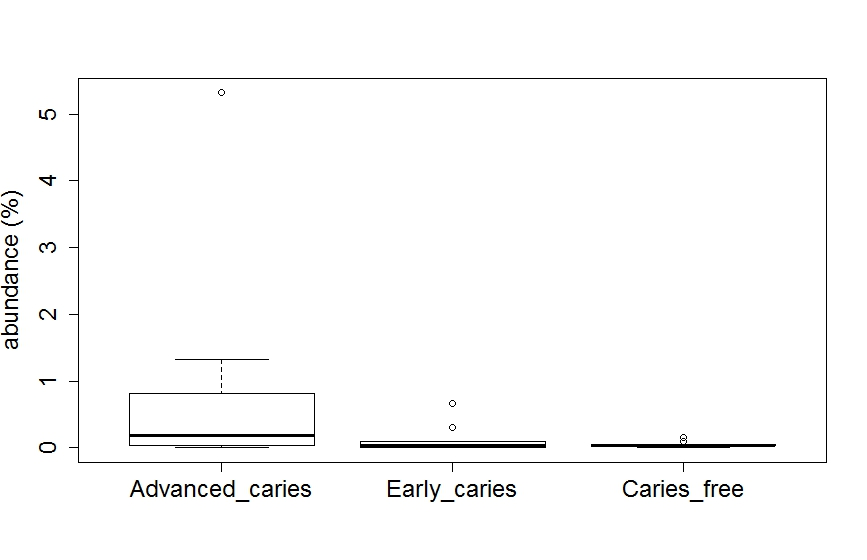
.

**Supplementary Figure 2.** Median relative abundance of *S. mutans* in the study groups. Differences between groups was significant by Kruskal-Wallis test (P ≤ 0.05). However, pairwise comparisons revealed a significant difference only in the AC-CF contrast. Detection rates were 100%, 80% and 90% for AC, EC and CF, respectively.


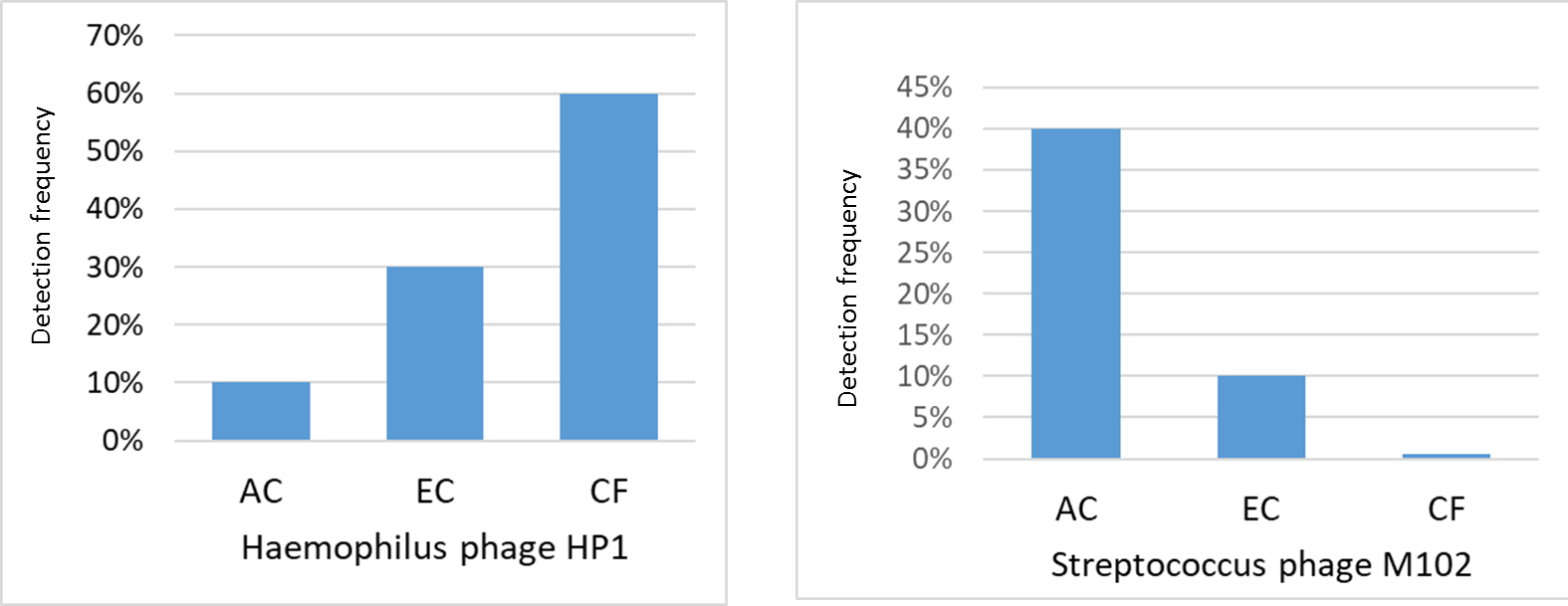


**Supplementary Figure 3.** Bacterial phages with significantly different detection rates between the groups; Chi-square test.

**
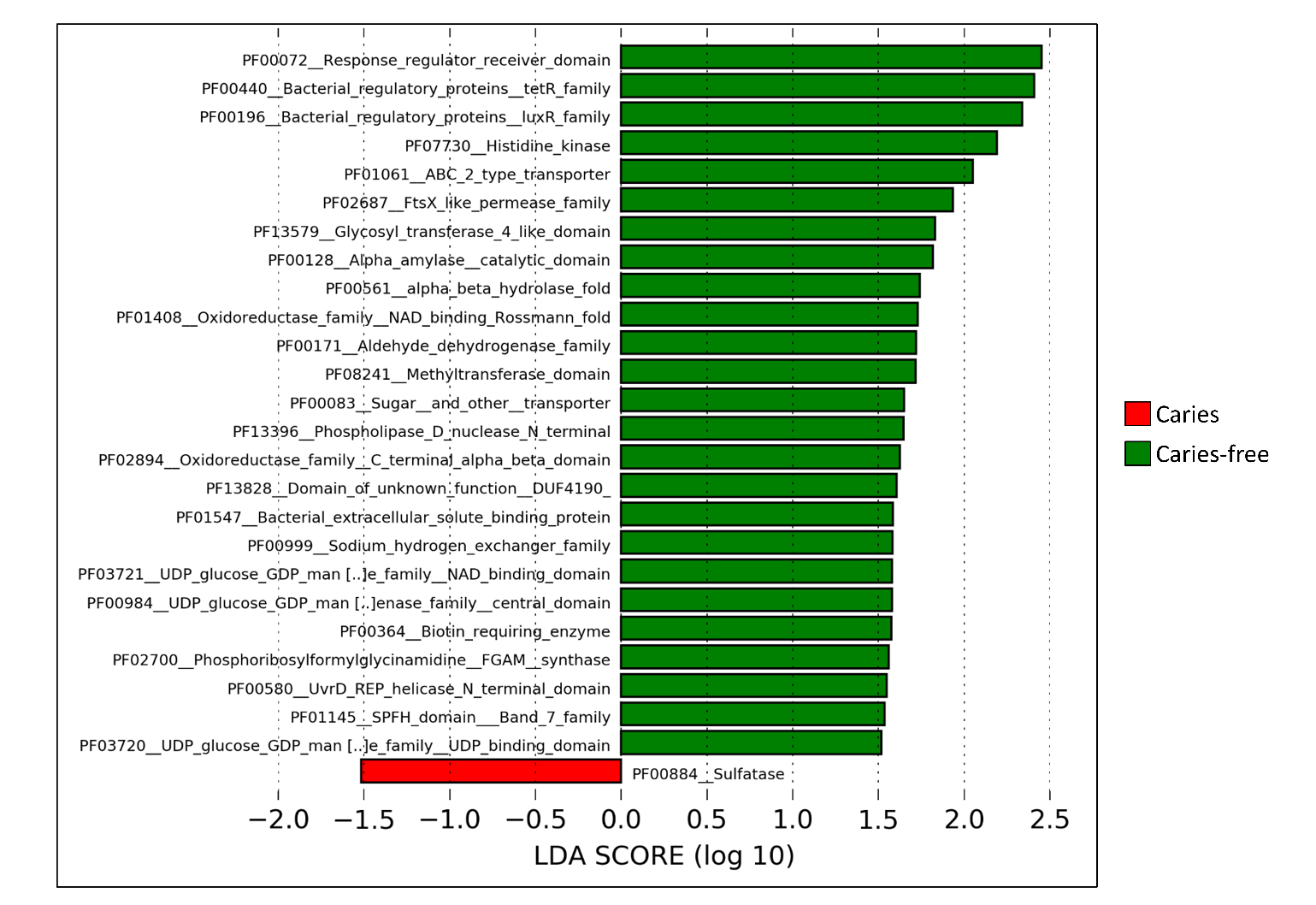
Supplementary Figure 4**. Differentially enriched protein families between the caries and caries-free groups, as identified by linear discriminant analysis (LDA) effect size analysis (LEfSe).

**Supplementary Files 1-9.** Provided as separate excel sheets (data sets).
